# Next-generation sequencing and comprehensive data reassessment in 263 adult patients with neuromuscular disorders: insights into the gray zone of molecular diagnoses

**DOI:** 10.1007/s00415-023-12101-6

**Published:** 2023-12-21

**Authors:** Martin Krenn, Matias Wagner, Gudrun Zulehner, Rosa Weng, Fiona Jäger, Omar Keritam, Merve Sener, Christof Brücke, Ivan Milenkovic, Agnes Langer, Dominic Buchinger, Richard Habersam, Katharina Mayerhanser, Melanie Brugger, Theresa Brunet, Maureen Jacob, Elisabeth Graf, Riccardo Berutti, Hakan Cetin, Julia Hoefele, Juliane Winkelmann, Fritz Zimprich, Jakob Rath

**Affiliations:** 1 Department of Neurology, Medical University of Vienna, Waehringer Guertel 18-20, 1090 Vienna, Austria; 2 Comprehensive Center for Clinical Neurosciences and Mental Health, Medical University of Vienna, Vienna, Austria; 3 Institute of Human Genetics, Klinikum Rechts Der Isar, School of Medicine, Technical University of Munich, Munich, Germany; 4 Institute of Neurogenomics, Helmholtz Zentrum München, Munich, Germany; 5 Department of Pediatric Neurology, Developmental Medicine and Social Pediatrics, Dr. Von Hauner’s Children’s Hospital, University of Munich, Munich, Germany

**Keywords:** Neuromuscular disease, Next-generation sequencing, Reanalysis

## Abstract

**Background:**

Neuromuscular disorders (NMDs) are heterogeneous conditions with a considerable fraction attributed to monogenic defects. Despite the advancements in genomic medicine, many patients remain without a diagnosis. Here, we investigate whether a comprehensive reassessment strategy improves the diagnostic outcomes.

**Methods:**

We analyzed 263 patients with NMD phenotypes that underwent diagnostic exome or genome sequencing at our tertiary referral center between 2015 and 2023. We applied a comprehensive reassessment encompassing variant reclassification, re-phenotyping and NGS data reanalysis. Multivariable logistic regression was performed to identify predictive factors associated with a molecular diagnosis.

**Results:**

Initially, a molecular diagnosis was identified in 53 cases (20%), while an additional 23 (9%) had findings of uncertain significance. Following comprehensive reassessment, the diagnostic yield increased to 23%, revealing 44 distinct monogenic etiologies. Reasons for newly obtained molecular diagnoses were variant reclassifications in 7 and NGS data reanalysis in 3 cases including one recently described disease-gene association (*DNAJB4*). Male sex reduced the odds of receiving a molecular diagnosis (OR 0.42; 95%CI 0.21–0.82), while a positive family history (OR 5.46; 95%CI 2.60–11.76) and a myopathy phenotype (OR 2.72; 95%CI 1.11–7.14) increased the likelihood. 7% were resolved through targeted genetic testing or classified as acquired etiologies.

**Conclusion:**

Our findings reinforce the use of NGS in NMDs of suspected monogenic origin. We show that a comprehensive reassessment enhances diagnostic accuracy. However, one needs to be aware that genetic diagnoses are often made with uncertainty and can even be downgraded based on new evidence.

**Supplementary Information:**

The online version contains supplementary material available at 10.1007/s00415-023-12101-6.

## Introduction

Neuromuscular disorders (NMD) constitute a wide range of conditions that affect motor neurons, peripheral nerves, the neuromuscular junction, and muscle tissue, making them highly heterogeneous both in terms of etiology and clinical presentation. A considerable proportion of these disorders are attributed to monogenic defects and the identification of the underlying cause is crucial for an accurate diagnosis, genetic counseling, and the development of targeted treatments [1].

Recent advancements in next-generation sequencing (NGS) technology have significantly improved diagnostic rates for various neurogenetic conditions [2]. With the use of NGS approaches such as multi-gene panels or whole-exome sequencing (WES), diagnostic yields of approximately 20–40% can be achieved in neurological disorders with a suspected monogenic cause, across different disease entities [2]. However, systematic data on the diagnostic application of NGS specifically in NMDs is limited. Previous studies have indicated a wide range of diagnostic yields, varying between 12.9% and 52.9%, primarily influenced by factors such as patient selection and the applied testing method [1, 3–9]. Hence, despite the use of comprehensive sequencing technologies, a significant number of individuals with NMDs remain undiagnosed, highlighting the need for continued efforts to improve the diagnostic rates for these disorders.

In general, a cost-effective strategy to increase the diagnostic accuracy is the systematic reanalysis of previously generated NGS datasets [10–12]. This may lead to higher diagnostic yields by incorporating new disease gene discoveries, variant reclassification, improved bioinformatic pipelines and additional phenotypic information becoming available over time [13]. Furthermore, the Clinical Genome Resource (ClinGen, https://clinicalgenome.org) publishes new recommendations for changes of the widely used variant interpretation criteria established by the American College of Medical Genetics and Genomics (ACMG) [14] on an ongoing basis.

The diagnostic benefits of a periodic NGS data reanalysis have been demonstrated for various neuropediatric phenotypes such as epilepsies and neurodevelopmental disorders [15–17]. While data on NMDs are still limited, a preliminary analysis of our cohort (including the first 72 individuals) has shown an increased diagnostic yield following a clinically oriented, interdisciplinary reassessment of WES data [4].

In our current follow-up study, we present the detailed clinical and genetic data from a large real-world cohort of 263 adult patients affected by NMDs. We report the initial results of diagnostic NGS, and in a further step, conduct a comprehensive reassessment by incorporating data from re-phenotyping, systematic NGS reanalysis and variant reclassification using the most recent ClinGen recommendations.

## Methods

### Patients

We retrospectively evaluated all patients of the Department of Neurology of the Medical University of Vienna, Austria, with a suspected NMD who underwent NGS (245 WES and 18 whole-genome sequencing (WGS)) between 2015 and 2023. All patients received standard clinical, electrophysiological, and imaging evaluation at the discretion of the treating physician. Genetic testing was carried out as part of the routine clinical work-up and indication was provided by consultant neurologists specialized in NMDs. Patients with an hereditary spastic paraplegia (HSP) phenotype were partly evaluated and referred to genetic testing by movement disorder specialists.

NGS was performed at the Institute of Human Genetics of the Technical University of Munich, Germany. We extracted clinical details as well as electrophysiological, imaging and laboratory data (including external genetic reports) from the patients’ records and updated phenotypic information prior to the reanalysis of NGS data. Patients were sub-grouped into five phenotype categories: 1) muscle disease (including suspected myopathies, myasthenic and myotonic syndromes), 2) neuropathies, 3) motor neuron disorders (MND), 4) suspected mitochondrial diseases, and 5) HSP. In case of overlapping manifestations, the leading clinical phenotype was used to classify patients (e.g., in patients with spastic paraparesis and additional mild neuropathy, the case would be classified as HSP). The ethics committee of the Medical University of Vienna approved the study (EC-Nr. 1201/2022 and 1021/2018).

### Next-generation sequencing

WES was performed using a SureSelect Human All Exon Kit (Agilent, 50 mb V5 or Agilent 60 mb V6) or a Twist Human Exome 2.0 Plus Comprehensive Exome Spike-in and Mitochondrial Panel for enrichment. Enrichment for WGS was performed with an Illumina DNA PCR-Free Library Preparation Kit. Sequencing was carried out on an Illumina HiSeq2500, HiSeq4000 or NovaSeq6000 system (Illumina, San Diego, California). Primary and secondary bioinformatic analysis was carried out using the exome variant analysis (EVAdb, https://github.com/mri-ihg/EVAdb) pipeline of the Institute of Human Genetics (Technical University of Munich, Germany) [18]. In detail, reads were aligned to the Genome Reference Consortium Human Build 37 (GRCh37) using Burrow–Wheeler Aligner [19]. For WES, we analyzed single nucleotide variants (SNVs) and small insertions and deletions using the SAMtools pipeline [20]. If the initial analysis was negative, a second analysis using the GATK HaplotypeCaller pipeline was carried out [21]. We used ExomeDepth and Pindel to detect copy number variations (CNVs) [22, 23] and analyzed mitochondrial DNA (mtDNA) using off-target reads, as described previously [24] and used a recessive filter for homozygous and compound heterozygous variants with a minor allele frequency (MAF) < 1%, carried out a phenotype-based filter with an Online Mendelian Inheritance in Man (OMIM) full-term search for variants with a MAF < 0.1% and assessed CNVs and mtDNA using a MAF filter < 0.01% and < 1%, respectively. WGS data were analyzed using GATK HaplotypeCaller only.

### Variant classification and reanalysis of NGS data

We reclassified all reported variants using the MANE Select transcript. We annotated variants according to the ACMG guidelines [14] with modifications proposed by ClinGen updates (https://clinicalgenome.org) and following the recommendations of the ACGS guidelines [25]. The REVEL score and SpliceAI were used for in silico predictions [26, 27]. We used additional constraint metrics from gnomAD (loss-of-function observed/expected upper bound fraction (LOEUF) and Z-scores) to evaluate the tolerance of genes against loss-of-function (LoF) and missense variants, respectively. Sanger sequencing of parental DNA was performed in 4 cases to confirm in *trans* occurrence. Additionally, we reanalyzed NGS data with initially reported negative results or variants of uncertain significance (VUS) including newly proposed NMD candidate genes (see supplementary table 1 for a list of the screened candidate genes that were not OMIM listed at the time of reanalysis between July 2022 and July 2023). The reanalysis was carried out in the same way as described above for the initial analysis. In terms of re-phenotyping, we performed a systematic chart review to incorporate new diagnostic findings (including laboratory, clinical neurophysiology and imaging data) that had become available in the meantime (i.e., between initial NGS testing and re-analysis).

### Statistical analysis

R (v4.3, 2023, R Foundation for Statistical Computing) and R Studio (v2023.6, 2023, RStudio PBC) were used for statistical analysis. The primary outcome was the diagnostic yield, defined as the occurrence of (likely) pathogenic variants in a disease gene compatible with the phenotype and the expected mode of inheritance. In case of compound heterozygous variants, both variants had to be classified as (likely) pathogenic to count as disease-causing. We analyzed the diagnostic yield for the entire cohort as well as for each predefined phenotype category. We used multivariable logistic regression to investigate whether clinical, demographic or methodical factors affected the likelihood of receiving a molecular diagnosis. Covariates included age at onset (in decades), gender, family history, phenotypic subgroups, NGS method (WES vs. WGS) and NGS approach (singleton, duo- or trio-NGS). Differences between continuous variables were compared using the Mann–Whitney U or t-test, and differences between categorical variables using the chi-square or Fisher’s exact test.

## Results

### Clinical and demographic cohort characteristics

We analyzed 263 patients (41% females, 59% males) with a median age of 49 years (IQR 38–60, total range 18–86). Most patients had a primary muscle disease (*n* = 80, 30%; median age 45, IQR 34–52), followed by MND (*n* = 72, 27%; median age 58, IQR 47–67), neuropathy (*n* = 50, 19%; median age 47.5, IQR 33–55), HSP (*n* = 47; 18%, median age 52, IQR 42.5–60.5) and suspected mitochondrial disease (*n* = 14, 5%; median age 40, IQR 37–47.5). The median onset of symptoms was in the fourth decade (IQR 3–6) and the majority of patients had a disease onset in adulthood (79%), while a positive family history (with at least one first or second degree relative being affected with a similar phenotype) was reported in 22%.

### Initial diagnostic yield of next-generation sequencing

WES was performed in 245 (93%) of these patients (96% singletons, 2% trio-WES, 2% duo-WES), and WGS in the remaining 18 (7%; 89% singletons, 11% duo-WGS) with a median coverage of 103 (IQR 89–127) and 52 (IQR 49–53) reads, respectively. The initial diagnostic yield of the laboratory was 20% (53/263 patients). In an additional 9% (23/263), a result of uncertain significance was reported. A dual molecular diagnosis was found in 1% (2/263) and secondary findings [28] in 2% (5/263).

### Diagnostic yield after variant reclassification

After the incorporation of new clinical and external genetic information as well as a reclassification of all previously reported variants using the ClinGen recommendations, we were able to solve 30% (7/23) of the cases with initial results of uncertain significance. 6% (3/53) of cases initially considered as “solved” were downgraded to “uncertain significance” and 9% (2/23) of uncertain results were reclassified as “unsolved” (see supplementary file 2 and 3 for variant details). Reasons for upgrading the 7 VUS to (likely) pathogenic variants were new phenotypic information in three patients (muscle biopsy results in two patients and biochemical analysis of 27-hydroxycholesterol levels in one patient with SPG5), a higher weighting of deleterious in silico predictions (i.e., ACMG PP3 criterion upgrade based on new ClinGen recommendation) in two patients, the combination of segregation and allelic information in one patient, and the combination of in silico data and confirmation of biallelic variant location in one patient. Among the initially solved cases, three were downgraded to "uncertain significance", including two patients with compound heterozygous variants in *FA2H*, with one being reclassified as a VUS. Additionally, one patient had a *TTN* variant, for which existing literature suggests no damaging effect based on functional studies [29]. The two cases with an initial result of uncertain significance that were reclassified as “unsolved” included one patient with a pathogenic *ANO5* variant and an intronic VUS that was reclassified as likely benign because of the presumed in *cis* status after Sanger sequencing of a parent. The second patient harbored a VUS in *SMCHD1*, and an external methylation status was normal, ruling out facioscapulohumeral muscular dystrophy type 2 (FSHD2).

### Reanalysis of cases with an initially negative NGS

Subsequently, we systematically reanalyzed all 210 patients who remained without a molecular diagnosis after initial NGS. The median time to reanalysis was 21 months (IQR 11–45, range 6–87). Reanalysis revealed (likely) pathogenic variants in disease-causing genes in three patients (1%) and VUS that were initially not reported in six patients (3%; see supplementary files 2 and 3 for variant details). The three newly solved cases included one patient with a typical autosomal recessive *RYR1*-associated myopathy, and a patient with chronic progressive external ophthalmoplegia and late-onset parkinsonism who had a likely pathogenic *POLG* variant. Both variants were either missed or not correctly attributed to the phenotype during the initial analysis. The third patient solved by reanalysis had a homozygous variant in *DNAJB4* for which a new gene-disease association was only recently discovered.

### First replication of recessive DNAJB4-related myopathy

A pathogenic homozygous frameshift variant in *DNAJB4* (NM_007034.5:c.308del) was found through reanalysis in a patient initially presenting with severe weight loss at the age of 32 followed by progressive respiratory failure due to diaphragmatic weakness, necessitating nightly non-invasive ventilation (see patient #60 in supplementary file 1 for a more detailed phenotypic description and a figure showing the muscle biopsy results). Homozygous *DNAJB4* variants resulting in a myopathy with early respiratory failure with highly variable symptom onset between age 1 and 45 years were only recently reported in four patients [30]. Functional studies indicated LoF as the underlying disease mechanism. Our patient has a strikingly similar phenotype with primary diaphragmatic weakness leading to respiratory failure and clinically only subtle myopathy confirmed by muscle biopsy. Thus, our case represents the first independent replication of autosomal recessive *DNAJB4*-related myopathy.

### Cases successfully resolved without NGS

Additional genetic testing to screen for variants not covered by our short-read NGS approaches was performed in 81 patients with the majority accounting for *C9orf72* repeat testing, which was negative in all 56 tested patients with a suspected MND. External testing solved 3 cases with a negative WES result (2 with spinal muscular atrophy (*SMN1*) and 1 with myotonic dystrophy type 2 (*CNBP*); NGS data of these cases were not re-analyzed). Supplementary table 2 provides results of external genetic tests.

Analyzing new clinical information that had become available after NGS analysis indicated an acquired (i.e., non-Mendelian) etiology in 6% (15/263) of cases. This included four patients with spinal lesions in whom HSP was initially suspected, five patients with immune-mediated conditions (i.e., neuropathies, myopathies or myasthenic syndromes) in whom genetic testing was performed to rule out a hereditary disorder and six patients with diverse causes ranging from leukemic muscle infiltration to delayed post-radiation bulbar palsy (see supplementary file 1 for detailed clinical information of all cases).

### Final diagnostic yield of NGS

**Fig. 1:**
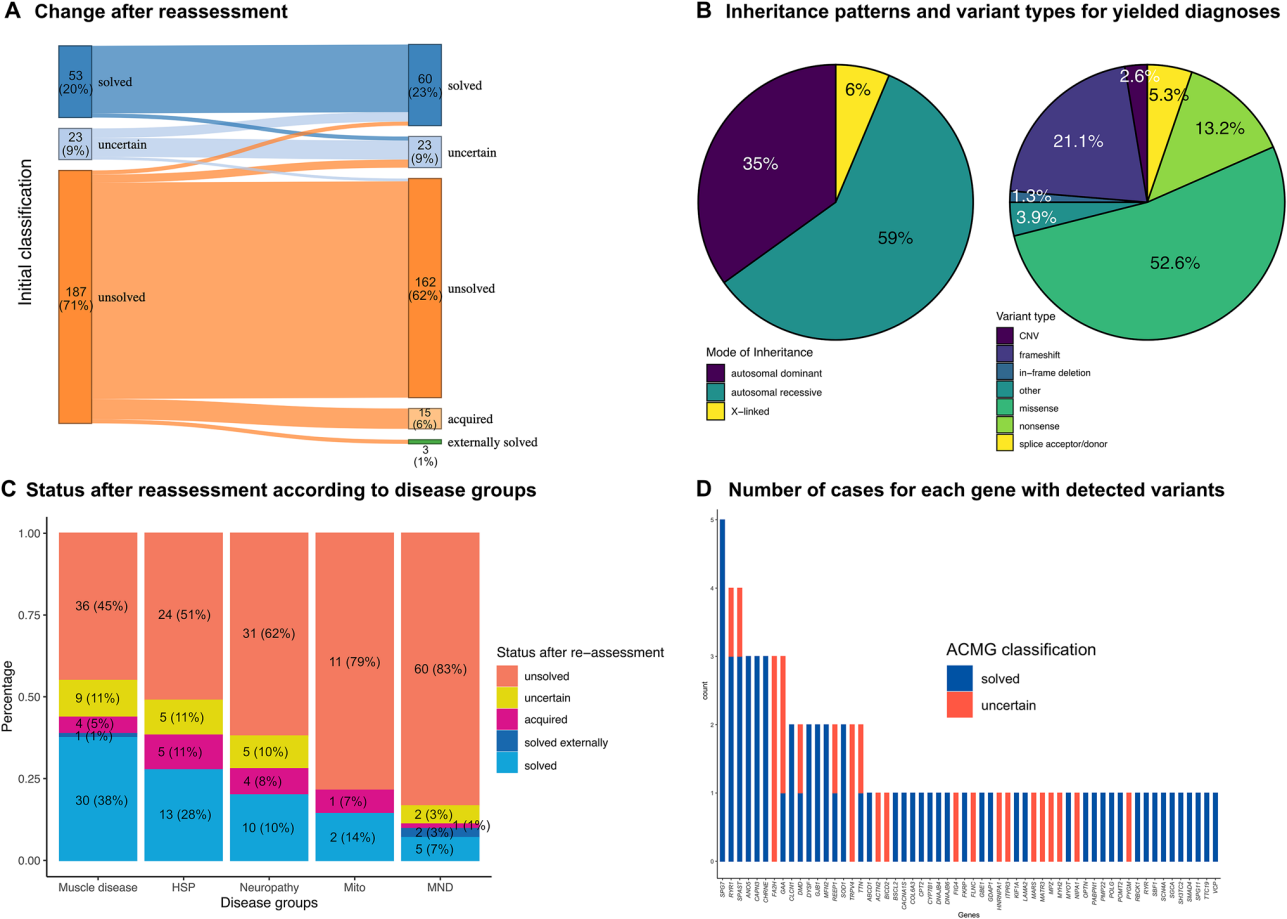
Overview of results after reassessment of NGS data. Panel A shows the change in classification of all patients after extensive reassessment including reclassification of all variants, reanalysis of all negative cases and extensive re-phenotyping and evaluation of all available clinical data including external genetic reports. Cases were regarded as solved if they had a (likely) pathogenic variant in a gene with an established gene-disease relationship compatible with the phenotype of the patient. In case of compound heterozygous variants both variants needed to be classified as (likely) pathogenic. Uncertain cases were patients where at least one of the variants in question was classified as a variant of uncertain significance (VUS). Cases were classified as having an acquired disease if NGS was negative and clinical and other diagnostic findings were compatible with a distinct non-monogenic disorder (see supplementary file 1 for a clinical description of all cases). Three patients were solved by external genetic testing after negative NGS. Panel B shows the inheritance pattern of all yielded diagnosis (excluding patients with VUS) and the type of all variants classified as (likely) pathogenic. Other variants included 1 intronic variant, 1 splice region (near-splice) variant and a GCG repeat expansion detected by WES in *ABPN1*. Panel C shows the classification of cases by disease (phenotype) groups after reassessment as described for panel A. Panel D shows a list of all genes with the number of detected (likely) pathogenic variants and VUS for each gene. ACMG denotes American College of Medical Genetics and Genomics, CNV copy number variation, HSP hereditary spastic paraplegia, Mito mitochondrial disease and MND motor neuron disease

**Fig. 2:**
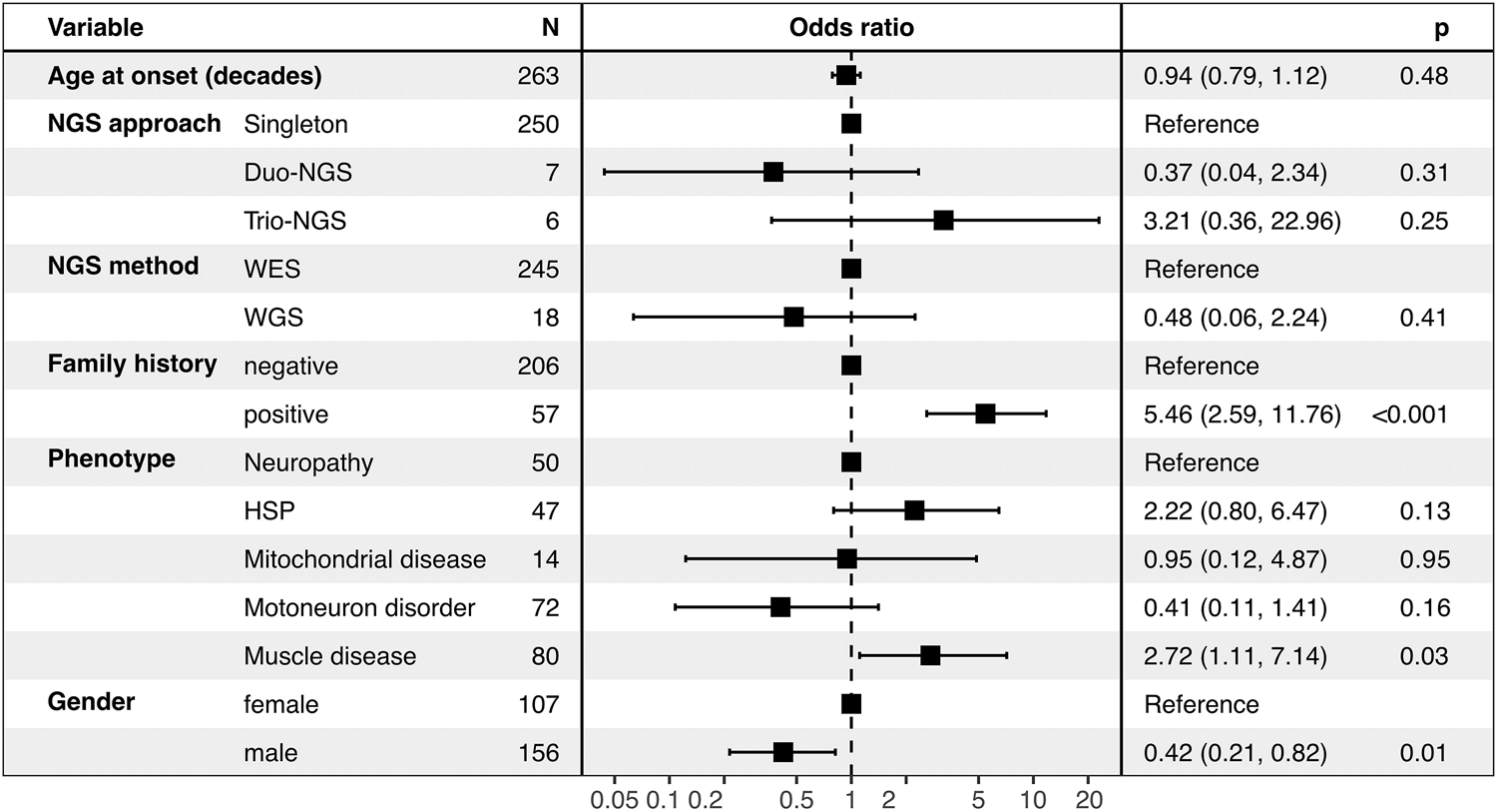
Forest plot. Odds for receiving a genetic diagnosis after reclassification and reanalysis of NGS data. Odds ratios are shown for per 1-unit increase for covariates included in the multiple logistic regression model. NGS denotes next generation sequencing, WES whole exome sequencing and WGS whole genome sequencing

After reclassification and reanalysis, a molecular diagnosis was ultimately established in 23% (60/263) of patients (Fig. 1), involving 44 distinct monogenic disorders. The highest yield was achieved in the muscle disease subgroup (38%; 30/80) and the lowest in patients with motor neuron disorders (7%; 5/72). Cases with a clinical suspected myasthenic syndrome, who were included in the muscle disease group because of the small number of patients, had a yield of 33% (3/9 patients). Figure 2 shows an overview of the diagnostic yields (overall and within the different phenotype subgroups) and variant characteristics. Table 1 shows clinical and sequencing details for all patients as well as a comparison between solved and unsolved cases. Supplementary file 2 (solved cases) and supplementary file 3 (cases with an uncertain diagnosis or cases reclassified as unsolved) provide an overview of all variants detected including detail variant annotation, population frequencies and in silico predictions.

**Table 1:** Baseline characteristics

	All patients (*n* = 263)	Patients with a molecular diagnosis (*n* = 60)	Patients without a molecular diagnosis (*n* = 203)	*p*-value
Median age (IQR) at NGS	49 (38–60)	48 (38–60)	49 (39–60)	0.64
Male sex	156 (59%)	28 male (47%)	128 male (63%)	0.03^§^
Disease group				< .001^§^
Neuropathy	50 (19%)	10 (17%)	40 (20%)	
Muscle disease	80 (30%)	30 (50%)	50 (25%)	
Motor neuron disorder	72 (27%)	5 (8%)	67 (33%)	
HSP	47 (18%)	13 (22%)	34 (17%)	
Mitochondrial disease	14 (5%)	2 (3%)	12 (6%)	
Decade of onset (median, IQR)	4 (3–6)	3 (1–5)	5 (3–6)	0.003^§^
Decade of onset by disease group (median, IQR)				
Neuropathy	4 (3–6)	3 (2–4)	5 (3–6)	0.50
Muscle disease	3 (1–5)	3 (1–4)	3.5 (2–5)	0.17
Motor neuron disorder	6 (5–7)	6 (5–7)	6 (5–7)	0.45
HSP	5 (3–6)	4 (4–6)	5 (3–6)	0.31
Mitochondrial disease	3.5 (1–5)	4 (2.5–5.5)	3.5 (1–4)	0.36
Disease onset in adulthood (≥ 18y)	208 (79%)	39 (65%)	169 (83%)	0.004^§^
Positive family history	57 (22%)	26 (43%)	31 (15%)	< .001^§^
NGS approach				0.76
Singleton	250 (95%)	56 (93%)	194 (96%)	
Duo	7 (3%)	2 (3%)	5 (3%)	
Trio	6 (2%)	2 (3%)	4 (2%)	

Clinical and sequencing details for all patients and comparison of patients with and without a molecular diagnosis after re-assessment of NGS data. The 3 patients solved by external genetic testing are included in the unsolved group. *HSP* denotes hereditary spastic paraplegia, *IQR* interquartile range and *NGS* next-generation sequencing

^§^statistically significant

### Factors associated with a molecular diagnosis

Multivariable logistic regression (Nagelkerke pseudo-R squared: 0.28) showed that male sex reduces the chance of receiving a diagnosis (odds ratio (OR) 0.42; 95%CI, 0.21–0.82). Moreover, patients with a positive family history were more likely to receive a diagnosis (OR 5.46; 95%CI 2.60–11.76). From a phenotypic point of view, the clinical suspicion of a myopathy was also associated with a positive diagnostic result (OR of 2.72 compared to the indicator group neuropathy; 95%CI 1.11–7.14). Other phenotype subgroups and NGS approach (WES or WGS; singleton, duo or trio) had no significant effect. In the univariate comparison, age of onset (in decades) was significantly lower in patients who received a diagnosis but this was no longer statistically significant in the multivariable model (OR 0.94, 95%CI 0.79–1.11).

## Discussion

Over the past decade, NGS has gradually entered clinical practice as a useful and cost-effective diagnostic tool [31]. Although neurogenetic conditions appear to be particularly suitable to NGS, it has been noted that patients with NMDs are still underrepresented in the literature [2]. Moreover, the majority of studies have focused on neuropediatric cohorts, and robust data on adults are scarce [32]. In an effort to bridge these gaps, we analyzed the diagnostic NGS findings in a large adult cohort including 263 individuals with NMDs. Secondly, we specifically aimed to assess the utility of a comprehensive reassessment pipeline incorporating data from NGS reanalysis, variant reclassification, re-phenotyping and external diagnostic tests.

The initial diagnostic yield of 20% across all phenotypes is largely concordant with previously published data on clinically mixed NMD cohorts [1, 3]. Some studies yielded significantly higher diagnostic rates, which may be explained by highly selected phenotypes [5, 9] or less stringent variant interpretation [33].

In a comparably small cohort, our group has demonstrated that the initially reported yield can be optimized by an interdisciplinary setting using a clinically oriented reassessment of NGS reports [4]. In the current study, we applied a more comprehensive and systematic strategy to further improve diagnostic accuracy. Thereby, the initial diagnostic rate could be increased by 3%, which is significantly lower than the 10% increase reported by a recent meta-analysis [11]. This discrepancy may be attributed to the literature being somewhat biased toward infantile and neurocognitive phenotypes with a higher rate of underlying monogenic defects. Additionally, we have downgraded variants previously classified as causative, which is a still underrecognized, albeit important approach to enhance diagnostic accuracy. We emphasize the importance of reassessing cases initially considered as solved, as variant downgrades may also have significant implications for genetic counseling, family planning or even therapeutic decisions.

Our initial interpretation of variants strictly followed the standards of the ACMG [14]. In our reclassification process, we additionally employed the updated ClinGen recommendations, which changes the weighting of multiple strands of evidence, including population frequency data, functional studies, and computational prediction tools, among others. This is particularly significant in adult neurological patients, as the absence of trio or segregation data may often hinder variant assessment, resulting in a high proportion of uncertain testing results.

In view of the above-mentioned findings, we consider it reasonable to periodically reassess NGS data of adult patients with NMDs, taking into account updated clinical and genetic information. Yet, we acknowledge that personal resources are often limited, making it unrealistic to provide such assessments for every single case as part of the diagnostic routine. To address this issue, we sought to identify predictive factors to aid in the preselection of individuals with a higher likelihood of receiving a molecular diagnosis. We found that patients with a positive family history or those presenting with a myopathy phenotype should be prioritized for a more elaborate work-up to uncover a monogenic cause. Moreover, we found female gender to be significantly associated with the detection of causative variants. The reason for this observation is not entirely clear. However, similar findings have been noted previously [34], and there is robust evidence that females require a higher variant burden to manifest with neurodevelopmental disorders [35]. Nonetheless, further research is warranted to gain a better understanding of this observed association.

A major strength of this study is that a comparably large and clinically representative real-world cohort was analyzed. Although already demonstrated for other disorders, we are, to our knowledge, the first group to investigate the utility of NGS data reanalysis in NMDs. Our work is further strengthened by in-depth phenotypic and molecular information available for all subjects with reported variants. We thus provide clinicians and geneticists with a valuable resource regarding variant- and gene-disease associations. Of note, all subjects underwent a comprehensive, unbiased sequencing approach (i.e., WES or WGS) which is advantageous over targeted gene panels [4]. For example, our presented *DNAJB4* case could only be resolved through an unbiased approach also accounting for preliminary gene-disease associations. We provide the first independent replication of recessively inherited *DNAJB4*-related myopathy with respiratory failure [30], which again emphasizes the scientific value of NGS, regardless of its diagnostic utility.

Our work also has some limitations. First, it is important to acknowledge the marked phenotypic heterogeneity of the investigated study population, which reflects the diverse range of patients encountered in a real-world clinical setting. As a result, only limited conclusions can be drawn from findings in smaller phenotypic subgroups. Given the retrospective nature of our study, there were no predefined clinical criteria to select patients for genetic testing. Although only specialized neurologists provided indications for NGS, we cannot exclude the influence of personal preferences and varying diagnostic thresholds. This may be particularly true for cases with subtle phenotypes, such as mild myopathies, that may be indistinguishable from functional disorders based solely on clinical grounds. Moreover, NGS is available to all patients in our institution if deemed clinically necessary by the treating specialist without economical restrictions or the need for prior targeted genetic analyses. This low threshold to perform NGS might partly explain the lower yield in our cohort. However, we believe the decreasing costs of NGS will lead to less stringent selection criteria and our cohort therefore is reflective of this trend.

Of note, the diagnostic yield in the neuropathy subgroup also needs to be interpreted with caution, as testing for *PMP22* (CMT1A) duplications is usually performed prior to NGS in our department, and the combined yield in an unselected neuropathy cohort would therefore most probably be higher. Similarly, the comparably low number of mitochondrial diseases in our cohort may be explained by targeted testing approaches in cases with a strong clinical suspicion. Further, mitochondrial disorders with a primary CNS phenotype (and additional neuromuscular features) did not fulfill our criteria for inclusion in this study.

Another inherent drawback of our study is the very low number of sequencing trios, which may have a negative impact on diagnostic outcomes [36]. NGS also has some technical limitations, making it particularly challenging to detect repeat expansions (myotonic dystrophies) or variants in genes with highly homologous pseudogenes (e.g., *SMN1* and *SMN2*). Finally, it is obvious that a large fraction of cases remains unsolved despite a comprehensive assessment. This may either be caused by non-Mendelian disease etiologies or by monogenic cases that remain unsolved due to methodological issues or thus far unidentified disease genes. Several efforts are already underway to overcome these obstacles, including RNA sequencing to account for transcript-level alterations [37], or optical genome mapping for a more accurate identification of structural variants [38]. Furthermore, long-read sequencing methods are becoming widely available, which enable more accurate sequencing of challenging regions in the genome such as repeat expansions and can provide phasing information [39]. Lastly, improvement of variant calling methods and the emergence of pangenomes will further improve clinical diagnostics in the future [40]. Considering these advances, we expect a further increase of diagnostic outcomes in the foreseeable future.

Overall, our results confirm the diagnostic utility of NGS in adult patients with NMDs. Our findings emphasize the importance of a periodic reassessment of genetic testing results based on both clinical and genetic information that becomes available over time. While the absolute increase in diagnostic yields may appear modest, such approaches are generally cost-effective and hold relevant implications for clinical decision making in individual patients. Ultimately, the findings of our study have the potential not only to enhance the diagnostic rates, but also improve patient outcomes in the long run, as an accurate molecular diagnosis is a prerequisite for the development of targeted therapies.

### Supplementary Information

Below is the link to the electronic supplementary material.

Supplementary file1 (DOCX 1272 kb)

Supplementary file2 (XLSX 30 kb)

Supplementary file3 (XLSX 24 kb)

## Data Availability

Data can be made available from the corresponding author (jakob.rath@meduniwien.ac.at) upon reasonable request and after approval from the ethics review board of the Medical University of Vienna.

## Abbreviations

ACMG: American College of Medical Genetics and Genomics
CNV: Copy number variation
IQR: Interquartile range
HSP: Hereditary spastic paraplegia
LoF: Loss of function
MAF: Minor allele frequency
MND: Motor neuron disease
mtDNA: Mitochondrial DNA
NGS: Next-generation sequencing
NMD: Neuromuscular disorder(s)
OMIM: Online Mendelian Inheritance in Man
OR: Odds ratio
SNV: Single nucleotide variant
WES: Whole-exome sequencing
WGS: Whole-genome sequencing
VUS: Variant(s) of uncertain significance
