# Supplementary material for: Next-generation sequencing and comprehensive data reassessment in 263 adult patients with neuromuscular disorders: insights into the gray zone of molecular diagnoses
Source: J Neurol. 2023 Dec 21;271(4):1937–46. doi: 10.1007/s00415-023-12101-6 (PMC10972933; doi:10.1007/s00415-023-12101-6)
Supplement: Supplementary file 1 — Supplementary file1 (DOCX 1272 kb) [file 415_2023_12101_MOESM1_ESM.docx]

SUPPLEMENTARY FILE

[**1**](#_heading=h.gjdgxs) **Clinical summaries 1**

[1.1](#_heading=h.30j0zll) Solved cases with pathogenic and likely pathogenic variants 1

[1.2](#_heading=h.1fob9te) Cases with variants of uncertain significance (VUS) 14

[1.3](#_heading=h.3znysh7) Cases in whom an acquired (non-monogenic) diagnosis was found after negative NGS. 19

[**2**](#_heading=h.2et92p0) **Supplementary tables 23**

[2.1](#_heading=h.tyjcwt) Screened candidate genes 23

[2.2](#_heading=h.3dy6vkm) External genetic testing 26

[2.3](#_heading=h.1t3h5sf) Sequencing details 26

# Clinical summaries

## Solved cases with pathogenic and likely pathogenic variants

**Patient #1** (*MFN2*): This male patient first presented at age 19 years due to gait disturbances and neuropathic pain in both lower extremities, especially when climbing stairs. Neurological examination revealed an increased muscle tone with brisk tendon reflexes in both legs. An MRI of the brain and the entire spine was unremarkable. Nerve conduction studies (NCS) were in line with an axonal-demyelinating sensorimotor polyneuropathy. The family history is negative.

**Patient #2** (*PABPN1*): This 61-year-old male patient initially reported mild dysarthria and dysphagia at 51 years of age. Symptoms were slowly progressive, and on examination, he showed mild proximal weakness in the lower limbs (MRC grade 4). CK levels were mildly elevated (200-400 U/l). Electrophysiological investigations indicated chronic neurogenic alterations, whereas a muscle biopsy rather pointed towards a myopathic cause (rimmed vacuoles and tubulo-filamentous inclusions).

**Patient #3** (*SPG7*): This male patient presented at 62 years of age with gait abnormalities and hypesthesia in both feet starting around the age of 56. Clinical examination showed a marked spastic paraparesis (MRC grade 3-4) with increased tendon reflexes and pyramidal tract signs. Neuroimaging was largely unremarkable, but diagnostic transcranial magnetic stimulation indicated a pyramidal tract lesion; NCS showed an axonal sensorimotor polyneuropathy. The family history is positive, as the patient's older sister was reported to have similar symptoms.

**Patient #4** (*RBCK1*): This woman initially presented with a childhood-onset dilated cardiomyopathy, requiring heart transplantation at 17 years of age. During childhood, she suffered from relapsing infections. In her 20s, she was diagnosed with acute febrile neutrophilic dermatosis (Sweet’s Syndrome). Moreover, she developed progressive muscle weakness, rendering her wheelchair-dependent. Neurological examination showed bilateral ptosis, proximally pronounced weakness of the lower limbs (MRC 2, pelvic girdle). Upper limbs were also, but less severely affected (MRC 4-, shoulder girdle). CK levels ranged between 80 and 1000 U/l. EMG showed a myopathic pattern. Muscle biopsy was inconclusive, showing fatty and fibrotic degeneration. The family history is unremarkable.

**Patient #5** (*SPG7*): This 65-year-old gentleman had experienced gait problems since the age of 39 years with a slow but continuous disease progression. Clinically, he displayed spasticity in both lower extremities with increased reflexes and *pes cavus*. MRI of the brain showed a cerebellar atrophy, an MRI of the spine was normal. Transcranial magnetic stimulation confirmed a pyramidal tract lesion. NCS were unremarkable. An ophthalmological assessment showed a bilateral partial optic atrophy. The patient's grandfather and brother are also affected.

**Patient #6** (*CAPN3*): This woman currently displays a limb-girdle pattern of muscle weakness and hyperCKemia since childhood, with clinical onset around 7 years old. By the age of 14, she required a wheelchair. On neurological examination in adulthood, she exhibited severe proximal weakness in all four limbs (MRC 0), with MRC 3-4 in distal muscle groups. Bulbar, facial, and ocular muscles were spared. Additionally, she has pulmonary dysfunction (reduced vital capacity), scoliosis, and contractures in the legs. She does not have cardiomyopathy. The patient's sister is also affected.

**Patient #7** (*SGCA*): This woman has a long history of a limb-girdle pattern of weakness since early childhood (around 3 years of age). Clinically, she has severe proximally pronounced weakness in upper and lower limbs (MRC 3) and no significant cardiac involvement. Ocular and bulbar muscles have always been unremarkable. CK was elevated around 1000 U/l. NCS were normal, EMG showed neurogenic changes, and a muscle biopsy was in line with a myopathy. The family history was negative with respect to neuromuscular issues.

**Patient #8** (*DMD*, *SCN4A*): This male patient has a history of slowly progressive muscle weakness in both legs with symptom onset around the age of 29 years. He has had difficulties walking (particularly stairs) ever since and is now wheelchair-dependent since the age of 50. In addition, the patient has a history of cold-induced myotonia, especially at freezing temperatures, but also with mild myotonic symptoms when bathing in cold water. Neurological examination showed a proximal muscle weakness in the lower limbs (MRC grade 2-3) with relatively preserved muscle strength in upper limbs. There were marked atrophies predominantly affecting gluteal and thigh muscles. Deep tendon reflexes were absent in the lower limbs. CK was elevated around 1000 U/l. Echocardiography confirmed a left ventricular hypertrophy with diastolic dysfunction and a muscle biopsy indicated a dystrophinopathy.

**Patient #9** (*MYOT*): This male patient experienced first symptoms around the age of 60 years, with progressive proximal weakness and atrophies in lower limbs. Moreover, he reported mild dysphagia. On examination, there was an asymmetric proximal weakness (left>right) with MRC grade 4- for knee extension and significant atrophies, especially in the quadriceps femoris muscle. Reflexes were diminished. At last follow-up, the patient had been able to walk unaided around 100 m. CK levels were constantly elevated at around 400 U/l. NCS showed an axonal sensorimotor polyneuropathy with chronic neurogenic alterations on EMG. The family history was possibly positive, as the patient's son also had elevated CK levels and myalgia (no genetic testing available).

**Patient #10** (*SPG7*): This woman first presented with gait disturbances at the age of 53. Neurological examination revealed vertical gaze palsy, dysarthria, mild ataxia, increased tendon reflexes in the lower limbs with spasticity, and positive pyramidal tract signs. MRI of the brain and spine yielded normal results. NCS only showed very subtle axonal changes. Transcranial magnetic stimulation indicated a central motor lesion.

**Patient #11** (*CHRNE*): This woman has experienced ocular symptoms since early childhood, primarily characterized by severely restricted eye movements and bilateral ptosis that worsen throughout the day. Additionally, she reported fluctuating dysarthria and mild generalized weakness. Repetitive nerve stimulation did not reveal a pathological decrement. Her ocular symptoms showed improvement with the use of 3,4-diaminopyridine and pyridostigmine. The family history was inconclusive.

**Patient #12** (*SPAST*): This woman has had a gait disorder since childhood. In addition, she has *pes cavus/equinus*. Clinically, she exhibits pronounced lower limb spasticity with brisk reflexes, positive pyramidal signs, and a spastic-ataxic gait pattern. In adulthood, symptoms progressed with frequent stumbling and falls. Moreover, she has urge bladder dysfunction with nycturia. Neuroimaging and neurophysiological investigations were normal. Reportedly, the patient's father had a similar type of gait disorder.

**Patient #13** (*TTN*): This woman first noticed bilateral foot drop at approximately 25 years of age, prompting a medical evaluation. Neurological examination confirmed pronounced muscle weakness in distal leg muscles, accompanied by significant calf atrophy. CK levels were elevated, measuring around 400 U/l. EMG results were non-specific, and NCS were entirely normal. Consistent with the clinical presentation, muscle MRI revealed fatty degeneration in the distal leg muscles, primarily affecting the soleus, extensor hallucis longus, and flexor digitorum longus muscles. A muscle biopsy of the soleus muscle was performed, but the results were inconclusive, showing fatty degeneration. There was no significant family history of similar symptoms.

**Patient #14** (*CHRNE*): This female patient presented with ocular symptoms since early childhood, primarily characterized by severely restricted eye movements and bilateral ptosis worsening throughout the day. She also reported fluctuating dysarthria and mild generalized weakness. A clinical examination additionally revealed a mild facial weakness. Repetitive nerve stimulation was normal. The family history is positive, as one brother also has bilateral ptosis.

**Patient #15** (*CPT2*): This female patient has reported exercise intolerance since childhood, accompanied by myalgia and muscle stiffness that can persist for days. Symptoms are alleviated by rest and carbohydrate intake. Due to her report of dark-colored urine, episodes of rhabdomyolysis were suspected. Neurological examination did not reveal any focal deficits. CK levels were not measured during symptomatic episodes. EMG results were normal.

**Patient #16** (*SPG11*): This woman presented with lumbar and lower limb pain at the age of 60. She underwent laminectomy, which provided only temporary relief. Additionally, she reported urge incontinence and dystonic head tremor. In retrospect, symptoms onset was around the age of 39 years. A neurological examination indicated a spastic paraparesis due to the presence of increased muscle tone, very brisk reflexes, and positive pyramidal signs in the lower limbs. Brain MRI revealed a mild atrophy in frontoparietal and cerebellar regions. Transcranial magnetic stimulation demonstrated significantly prolonged latencies, indicating a pyramidal tract lesion.

**Patient #17** (*PMP22*): This gentleman currently experiences cramps in his hand muscles, particularly when exposed to cold temperatures, a symptom that has been present since adolescence. Around the age of 50, obvious muscle atrophy and reduced fine motor skills were observed. He also suffers from hearing loss. Clinically, he demonstrates mild proximal weakness (MRC grade 4) in both upper and lower limbs, along with more pronounced distal weakness and significant atrophy (MRC grade 2 in hand muscles, 2-3 for foot extension and flexion). As a result, he exhibits a stepping gait. He also reported cold-induced paresthesia but no other sensory abnormalities. NCS showed a primary demyelinating polyneuropathy. EMG showed (mainly chronic) neurogenic alterations. The patient's daughter is also affected by the condition.

**Patient #18** (*GDAP1*): This woman has been diagnosed with hereditary motor neuropathy since early childhood (at 3 years of age). There is a positive family history for the disease, with known consanguinity and two cousins exhibiting similar phenotypes. In an examination during adulthood, severe distal muscle weakness was observed, with an MRC grade of 0 for finger and foot flexion and extension, grade 2 for wrist movements, grade 4 for elbow and shoulder, grade 2 for knee, and grade 3 for hip movements. Additionally, she has recurrent laryngeal nerve palsy. NCS revealed signs of demyelination. In addition to hereditary motor neuropathy, she developed autoimmune myasthenia gravis in adulthood, which was confirmed by the presence of acetylcholine receptor antibodies (AChR-Ab).

**Patient #19** (*SPAST*): This male reported slowly progressive gait problems since the age of 35 years with occasional falls. Clinically, he displayed a spastic gait disorder with increased muscle tone, brisk reflexes and pyramidal signs in the lower limbs. There is also a mild weakness in both legs (MRC 4). Both NCS and transcranial magnetic stimulation were normal. Neuroimaging data are not available. A cousin had similar symptoms.

**Patient #20** (*DYSF*): This woman has been experiencing progressive muscle weakness in all four limbs since the age of 32, resulting in gait problems and recurrent falls. Upon examination, she presented with predominantly lower extremity proximal tetraparesis, with an MRC grade of 3 in the upper limbs and grade 1-2 in the lower limbs, along with absent tendon reflexes. CK levels were elevated at 600-700 U/l. EMG revealed neurogenic changes and signs of myotonia. NCS showed no significant abnormalities. The patient reported consanguinity, but no other family members exhibited comparable symptoms.

**Patient #21** (*MFN2*): The male patient reported a progressive muscle weakness since adolescence primarily involving distal muscle groups of the lower limbs. Over time, symptoms extended to more proximal muscles and upper limbs. At the time of genetic testing, he was able to walk with one stick. Clinically, he had marked bilateral *pes cavus*. Muscle weakness was distally pronounced with MRC grade 4 in hand muscles and grade 3 for foot flexion and extension. Deep tendon reflexes were absent. There were no overt sensory abnormalities. However, NCS showed a marked axonal-demyelinating sensorimotor polyneuropathy. EMG reflected the neurogenic changes. The family history is positive with the patient's mother and brother being similarly affected.

**Patient #22** (*ANO5*): In this gentleman, asymptomatic hyperCKemia (range 2000-6000 U/l) was found at 57 years of age. Subsequently, an atrophy of thigh muscles was recognized, leading to a further evaluation. Over the past years, symptoms progressed and he has developed a weakness (MRC grade 4 for elbow flexion, hip flexion and abduction, knee extension and foot dorsiflexion, right>left). At the last follow-up visit, he used 1-2 walking sticks.

**Patient #23** (*RYR1*): This woman has experienced non-progressive neuromuscular issues since birth/early childhood. On clinical examination, she has a mild tetraparesis (MRC 4) with absent reflexes. In addition, she has severe scoliosis and contractures. Anesthesia in childhood led to an episode reminiscent of malignant hyperthermia. CK was elevated up to 3300 U/l. EMG showed myopathic changes. The grandfather and the mother have a similar phenotype.

**Patient #24** (*CLCN1*): In this male patient, symptoms including muscle stiffness and exertional generalized weakness have been present since birth. Besides a mild generalized weakness, he shows a positive lid lag phenomenon with myotonia affecting facial muscles as well as arm and leg muscles with a positive warm-up phenomenon, but no percussion myotonia. Myotonia could be confirmed by EMG. The brother was reported to be severely disabled since birth.

**Patient #25** (*SBF1*): This gentleman reported vertigo, tinnitus and hearing impairment since the age of 35 years. Gait disturbances with unsteadiness had developed before his 50^th^ birthday. A clinical examination revealed a downbeat nystagmus, cerebellar dysartria, rest tremor with marked ataxia, distal sensory impairment (reduced vibration sense) and areflexia. CK was elevated (800-1600 U/l). NCS showed a primary axonal sensorimotor polyneuropathy. Neuropsychological testing indicated cognitive impairment, especially with regard to processing speed. An MRI of the brain was normal.

**Patient #26** (*GJB1*): This female patient reported onset of symptoms at 34 years with a weakness for toe extension. She also has neuropathic pain in both lower limbs. Clinical examination showed normal strength in upper limbs, but distal weakness in lower limbs, mainly affecting foot dorsiflexion (MRC 2), accompanied by absent vibration sense. She also has bilateral *pes cavus*. In NCS, no potentials could be derived, indicating severe sensorimotor polyneuropathy. Her father has had similar symptoms since the age of 40 years.

**Patient #27** (*SPG7*): This male patient presented with a slowly progressive spastic gait first noted shortly after this 30^th^ birthday. On examination, he exhibited mild paraparesis with increased deep tendon reflexes. Gait was spastic-ataxic. Brain MRI revealed a cerebellar atrophy, imaging of the cervical spine was normal. NCS and EMG was normal, but transcranial magnetic stimulation revealed a central lesion (located below the cervical spine). The family history was negative.

**Patient #28** (*COL6A3*): This gentleman had a history of muscle weakness affecting all four limbs first noted at 28 years of age. This resulted in difficulties climbing stairs and carrying weight. CK levels ranged between 700 and 1000 U/l. Neurological examination showed a mild weakness for elbow flexion and extension bilaterally (MRC 4). There were no significant atrophies. Both EMG and a muscle biopsy indicated a moderate myopathic syndrome with dystrophic features. The family history was unremarkable.

**Patient #29** (*ANO5*): In this female patient, early psychomotor development was delayed, and she experienced recurrent episodes of hyperCKemia in childhood/adolescence. Neurological examination in adulthood showed a mild, diffuse weakness in all limbs (MRC 4-5). In spite of relatively preserved muscle strength, the patient has become wheelchair dependent, suggesting a non-organic component. She also has polyarthritis and multiple psychiatric issues. EMG showed mild myopathic changes. CK was elevated up to 5000 U/l. The family history was negative for neuromuscular symptoms.

**Patient #30** (*TTC19*, *TNFRSF13B*): This woman had experienced neurodevelopmental delay and behavioral issues early in childhood. In adulthood, she developed severe dysphagia, mutism, ataxia, and spastic tetraparesis rendering her wheelchair dependent. Deep tendon reflexes were absent. Brain MRI showed bilateral hyperintensities in the white matter (periventricular, basal ganglia) and cerebellar atrophy. The findings were found to be consistent with a mitochondrial disorder. In addition, she had intractable epilepsy (with recurrent status epilepticus), polyneuropathy and IgA deficiency.

**Patient #31** (*SOD1*): This female patient started to experience foot drop at the age of 47 years. Symptoms started unilaterally but progressed to the contralateral foot, and at the same time, she also noted a weakness in the right hand. Due to frequent falls, she requires walking sticks. On examination, she had a distal weakness in both upper and lower limbs (left>right). Upon initial assessment, cranial nerves were normal and there were no upper motor neuron signs. Overall, symptoms have been only slowly progressive over several years. EMG revealed fasciculations/fibrillation in 3 out of 4 levels. MRI of the neuroaxis did not show any relevant abnormalities. The patient's mother also had an early-onset ALS phenotype.

**Patient #32** (*OPTN*): First symptoms were reported at the age of 40 when this male patient initially experienced dysphagia, lethargy, and headaches. Subsequently, he also developed weakness in the right arm and mutism. Furthermore, he exhibited behavioral abnormalities including apathy and episodes of aggressive behavior. Clinically, he was diagnosed with frontotemporal dementia/motor neuron disease overlap. An MRI of the brain showed FLAIR hyperintensities in the pyramidal tract. EMG results showed signs of denervation in 3 out of 4 levels. The condition progressed rapidly, and the patient passed away at the age of 41. The histological workup revealed a marked TDP-43 proteinopathy involving upper and lower motor neurons.

**Patient #33** (*RYR1*, *CACNA1S*): This female patient reported first symptoms around the age of 39 years with weakness in both upper limbs, subsequently also involving the legs (including weakness, muscle cramps, muscle pain). At this time, a muscle biopsy was performed and the caffeine-halothane contracture test was positive, confirming malignant hyperthermia. Clinical examination revealed a bilateral, proximal upper limb weakness (MRC grade 4) with normal deep tendon reflexes. CK levels were elevated between 500 and 1000 U/l. EMG indicated myopathy, NCS were normal. An MRI of pelvic girdle muscles was unremarkable. The patient's sister and daughter also had a history of MH.

**Patient #34** (*FKRP*): At 27 years of age, this female patient had noted symmetric weakness in both legs, leading to difficulties, e.g., when climbing stairs. Symptoms were slowly progressive, and she remained able to walk without aid for 17 years after symptom onset. On examination, she displayed a proximally pronounced weakness of all four limbs (MRC 3-4). CK is mildly elevated at around 300 U/l. EMG was myopathic, no muscle biopsy was performed. It is noteworthy that the patient also had a history of stroke (basilar artery occlusion). The family history was negative with respect to neuromuscular conditions.

**Patient #35** (*ANO5*): This gentleman had first noted mild diffuse muscle weakness around 40 years of age (was not able to run any more). This became more evident 6 years later, when he also developed pain in the gluteal area as well as gait instability. Neurological examination showed a proximal weakness in the lower limbs (MRC 4) with absent tendon reflexes. CK was elevated at 1000-2000 U/l. EMG showed mild myopathic alterations. An MRI of the heart did not show a cardiac involvement. The patient's mother also had gait problem without a definite diagnosis.

**Patient #36** (*DYSF*): This female patient has a progressive muscle weakness since the age of 24, mainly affecting distal leg muscles. CK levels have already been elevated up to 8000 U/l before symptom onset and have remained high ever since. Clinical neurological examination revealed a distally pronounced muscle weakness mainly in lower limbs with MRC grade 4 for knee extension and 3-4 for foot dorsiflexion, resulting in a stepping gait. Significant atrophies were found in calf muscles. EMG showed mild neurogenic changes. Muscle MRI showed significant atrophies and fatty degeneration of lower limb muscles sparing the popliteus muscle, which is concordant with a dysferlinopathy. The patient's older brother is also affected by the condition.

**Patient #37** (*BSCL2*): This male patient first noticed weakness in his right hand when he was 58 years old. Three years after the onset, the left arm was affected in a similar manner. A clinical examination led to a diagnosis of flail-arm syndrome. Clinically, he exhibited severe proximal weakness in both arms (MRC 1-2 for shoulder abduction) and predominantly left-sided distal weakness (MRC 0-1 on the left, MRC 4 on the right hand). Proximal muscles in the legs were spared, while foot dorsiflexion was markedly impaired (MRC 1). Significant atrophy was observed, but no bulbar or upper motor neuron signs were present. NCS showed a severe motor polyneuropathy (axonal > demyelinating). There was no family history of motor neuron disease.

**Patient #38** (*SMDA4*): This female patient reported weakness with atrophies in both hands occurring around 24 years of age. Neurological examination confirmed a bilateral ulnar nerve palsy. NCS was in line with a bilateral (right > left) primary axonal lesion of the ulnar nerve and carpal tunnel syndrome. Nerve ultrasound confirmed massive thickening of both nerves (ulnar > median). MRI of the brain and brachial plexus were normal. In addition, she has facial dysmorphism, brachydactyly, obesity and a small stature. Heart MRI indicated diffuse fibrosis. Family history was negative/inconclusive.

**Patient #39** (*REEP1*): This male patient described a progressive gait disorder since the age of 69. A neurological exam revealed brisk deep tendon reflexes throughout and spasticity in both lower limbs. Gait was spastic-ataxic. He also had pyramidal tract signs and reduced vibration sense. NCS showed a sensorimotor, axonal and demyelinating polyneuropathy. Transcranial magnetic stimulation indicated an additional central lesion. MRI of the brain and spine showed no specific abnormalities.

**Patient #40** (*GAA*): This man has a progressive muscle weakness since the age of 50, predominantly affecting the shoulder and pelvic girdle. On examination, he has proximal atrophies in arms and legs with symmetric weakness (MRC 4). NCS were largely normal, EMG was mildly neurogenic. CK was mildly elevated (around 300 U/l). Biochemical (enzyme) testing was in concordance with Pompe disease. The patient had experienced an episode of pneumonia with severe dyspnea requiring ventilation. The patient's brother succumbed to pneumonia.

**Patient #41** (*SPAST*): This male developed gait and balance disturbances at the age of 59. He was initially diagnosed with amyotrophic lateral sclerosis. The condition has progressed slowly with the patient remaining able to walk unaided 10 years after onset. Neurological examination revealed no circumscribed muscle weakness, but marked spasticity and brisk reflexes in the lower extremities. Brain MRI showed mild microangiopathic alterations, spinal MRI did not show any signs of myelopathy. Routine CSF analysis was unremarkable. Transcranial magnetic stimulation was normal. The family history was negative.

**Patient #42** (*SOD1*): This male patient experienced symptom onset at 69 years of age. Initially, he had a distally pronounced weakness in the left lower limb. Few months later, the right leg was also affected, leading to recurrent falls. He also reported reduced fine motor skills in both hands. Clinically, he had paraplegia and distally pronounced, relatively symmetric weakness in both upper limbs (MRC 4 proximally, MRC 3 distally) with marked atrophies and brisk reflexes. Brain MRI showed frontal, thalamic and cerebellar atrophies, characteristic signal alterations in the corticospinal tract as well as microangiopathic changes. Muscle ultrasound confirmed fasciculations in 3 out of 4 levels. The patient's father was also affected and died 3.5 years after onset.

**Patient #43** (*POMT2*): This male had symptom onset in early childhood (around 2 years) with progressive problems sitting and walking, eventually requiring a wheelchair in his 30s. In adulthood, he exhibited a severe proximal tetraparesis (proximal MRC 1-2, distal MRC 4-5) with atrophies. A muscle biopsy showed a myopathic pattern. The family history was normal.

**Patient #44** (*CHRNE*): This male patient has experienced ophthalmoparesis since childhood and generalized weakness since the age of 26 years. Pyridostigmine had a positive effect on ptosis, whilst salbutamol was overall ineffective. The family history was unremarkable.

**Patient #45** (*SH3TC2*): This woman has a gait disorder since the age of 26 years. CK was around 500 U/l. Neurological examination showed a lower extremity-predominant weakness (MRC 0 for foot extension and dorsiflexion, MRC 4 for knee extension, MRC 4 in arms/hands) with pallhypesthesia. NCS showed high-grade demyelinating, sensorimotor polyneuropathy with neurogenic changes on EMG. CSF analysis was normal. The cauda equina shows contrast enhancement on MRI. Nerve ultrasound showed moderate thickening of peroneal and tibial nerves. The family history was negative.

**Patient #46** (*ABCD1*): This male patient has gait problems since the age of 49 with significant progression over the past 3 years. Intermittently, he has exertion-dependent pain in both thighs. A neurological exam showed a mild paraparesis (MRC 4 on both sides) with increased deep tendon reflexes, mild spasticity and pyramidal tract signs. MRI of the brain showed white matter lesions in the mesencephalon and cerebellum. Spinal MRI was largely normal. NCS revealed a demyelinating polyneuropathy, while transcranial magnetic stimulation also pointed towards an additional central motor lesion. One brother seems to be similarly affected, but no genetic test result is available.

**Patient #47** (*CLCN1*): This female patient has myotonic symptoms (muscle cramps, difficulties opening after fist closure, etc.) worsening with temperature changes and predominantly affecting her hands. Symptoms onset was approximately around the age of 12 years. CK was elevated around 1000 U/l. EMG showed myotonic discharges. The family history was unremarkable with regard to neuromuscular phenotypes.

**Patient #48** (*SPG7*): This female had reported a slowly progressive unstable gait starting at the age of 56 years leading to falls several times per year. Moreover, she has urinary incontinence and optic atrophy. Clinically, she has a spastic muscle tone with brisk reflexes in the lower extremities (left > right) and mild bradykinesia in the left hand. Gait was spastic-ataxic. The MRI of the brain and spine showed no structural correlate. NCS was normal, but transcranial magnetic stimulation pointed towards a central motor lesion. Family history was negative.

**Patient #49** (*DNAJB6*): The female has experienced proximal weakness in her lower limbs since the age of 62, resulting in difficulties climbing stairs and standing up from a sitting position. More recently, she developed pain in both thighs. Her arms, hands, and bulbar muscles were largely unaffected. Upon examination, bilateral lower extremity weakness with atrophies was observed (MRC 4 in proximal, 4- in distal leg muscles). Additionally, her gait showed a stepping pattern with a positive Trendelenburg sign. Muscle MRI showed a severe fatty atrophy in gluteal and proximal calf muscles. EMG was in line with myopathy/myositis. The family history was negative.

**Patient #50** (*VCP*): The male individual has described gait problems due to lower limb weakness and stiffness since age 64. CK was elevated up to 400 U/l. NCS were normal. EMG showed chronic neurogenic changes, fibrillations and positive sharp waves. Muscle ultrasound showed fatty degeneration, especially in the gastrocnemius muscle and rare fasciculations in upper and lower limbs. Initially, a motor neuron disease was suspected.

**Patient #51** (*KIF1A*): This female patient had motor delay since birth and developed spastic gait problems at the age of 5 years, eventually rendering her wheelchair-dependent at the age of 15. Neurological examination in adulthood showed a left third cranial nerve palsy, bilateral upper limb ataxia with moderate weakness (MRC grade 4) and severe weakness (MRC grade 2-3) and spasticity of the lower limbs with a positive Babinski sign. Deep tendon reflexes of the lower limbs were brisk. Sensation for light touch was reduced in the legs. She also suffered from spastic bladder outlet obstruction. NCS demonstrated an axonal neuropathy of the lower limbs. A minimal atrophy of the lateral part of the cerebellar hemispheres was detected on an MRI scan of the brain, whereas the spinal cord appeared morphologically normal. In addition, she suffered from antiphospholipid antibody syndrome with relapsing episodes of deep vein thrombosis and myocardial infarction. She died at 40 years of age due to recurrent thromboembolic complications. The patient's sister is also affected with spastic paraparesis.

**Patient #52** (*RYR1*): The female patient has proximal muscle weakness since her first year of life. She is able to walk unaided but has difficulties standing up from a sitting/squatting position and when climbing stairs. Examination showed a vertical gaze palsy, a mild proximal weakness in both upper and lower limbs (MRC 4) with distally preserved strength and absent/reduced tendon reflexes. CK was mildly elevated. Muscle MRI revealed severe fatty degeneration of gluteal and ischiocrural muscles. NCS were normal, EMG showed neurogenic changes. Family history was negative.

**Patient #53** (*CYP7B1*): This female patient has a slowly progressive gait disorder since her first decade of life with frequent stumbling. She requires a wheelchair since her 35^th^ birthday. Additionally, she has dysphagia. In adulthood, she had spastic paraparesis (proximal MRC 3, distal MRC 0-1 for foot dorsiflexion) with increased reflexes and pyramidal signs. Brain MRI showed unspecific white matter hyperintensities. MRI of the spinal cord showed no structural correlate. NCS showed an axonal sensorimotor polyneuropathy. Spasticity was treated with botulinum toxin and a Baclofen pump. Family history was negative.

**Patient #54** (*GJB1*): This woman has been reporting distal muscle weakness with pronounced sensory abnormalities since her 30s. The neurological examination revealed distal muscle weakness (MRC 4) with bilaterally absent ankle jerk reflex, as well as marked sensory impairment affecting light touch and joint position sense. NCS indicated a demyelinating polyneuropathy. She previously tested negative for CMT1A. Additionally, the patient's sister was also affected by similar symptoms, while her son has *pes cavus* but has not undergone genetic testing.

**Patient #55** (*GBE1*): This female patient presented with progressive weakness starting at the age of 58, initially involving the right arm. Four years later, the left arm was also affected. Examination showed a marked proximal weakness in upper limbs (MRC 2 shoulder abduction on the right, MRC 4 on the left side). Lower limbs were less severely affected (MRC 4 on both sides). Sensory examination was normal. Based on clinical findings, a motor neuron disease was suspected. She further developed dyspnea and dysphagia. CK was elevated (300-650 U/l). NCS was normal. EMG showed chronic neurogenic changes, fasciculations and positive sharp waves. Muscle ultrasound confirmed fasciculations in 2 out of 4 levels (cervical/lumbar). Transcranial magnetic stimulation did not indicate a central lesion. MRI of the brain and cervical spinal cord was largely unremarkable. The family history was negative.

**Patient #56** (*CAPN3*): This gentleman has experienced motor developmental delay becoming evident in early childhood years. Over short distances, he was able to walk unaided and ride a bicycle in adolescence and young adulthood. He constantly requires a wheelchair since age 48. On neurological examination, he displays severe tetraparesis (proximal MRC 1, distal MRC 4-5) with reduced tendon reflexes. In adolescence, CK levels were elevated around 1000 U/l. EMG was myopathic. Muscle biopsy pointed towards a muscular dystrophy with an absent 94 kDa band in Calpain-3 Western blot analysis. Family history was unremarkable with regard to neuromuscular symptoms. One uncle was reported to have multiple sclerosis.

**Patient #57** (*LAMA2*): This male patient has noted a progressive proximal muscle weakness since age 30. Clinically, he had a proximally pronounced tetraparesis (MRC 4). Both CK was elevated (900 U/L). NCS were normal. EMG and muscle biopsy primarily showed myopathic changes. Muscle MRI revealed marked atrophies, mainly affecting posterior muscle groups (semitendinosus, semimembranosus, biceps femoris muscles). An MRI of the brain showed bilateral symmetric leukoencephalopathy. The family history was negative.

**Patient #58** (*RYR1*): This man has mild muscle weakness since early childhood. Activities of daily life are only mildly impaired. A neurological examination showed facial weakness, dysarthria/dysphonia and mild proximal weakness (MRC 4) with deep tendon reflexes being absent throughout. CK was elevated at 350 U/l. NCS was normal, EMG was neurogenic. By contrast, muscle histology was in concordance with a myopathy. Cardiological check-ups were normal. The family history is positive, as 4 out of 7 siblings of the patient are similarly affected.

**Patient #59** (*POLG*): This female patient had gait imbalance with oscillopsia and slowed speech since the age of 62 years. On clinical examination, she had vertical gaze palsy, right-sided ptosis, reduced fine motor skills, apraxia, mild rigidity and brisk reflexes throughout. Later on, the patient developed dysarthria and dysphagia. MRI of the brain showed mild temporal atrophy. Routine CSF was normal. VOG indicated a cerebellar pathology. DAT-SPECT showed a nigrostriatal degeneration. She died at 70 years of age.

**Patient #60** *(DNAJB4):* This female patient first noted unvoluntary weight loss at the age of 32. Approximately one year later she experienced progressive breathing difficulties during exertion which ultimately led to respiratory insufficiency requiring non-invasive ventilation (NIV) during the night. A fluoroscopy showed limited movement of the diaphragm on both sites during inspiration and expiration. Spirometry revealed a restrictive pattern, a chest CT showed no sign of interstitial lung disease and a heart ultrasound was normal. A neurological examination showed generalized muscle atrophy, cachexia, pectus excavatum but no muscle weakness. CK was in the normal range. An initial externally performed muscle biopsy of the quadriceps femoris muscle revealed disseminated atrophic fibers without angular appearance and necrotic fibers at different stages of regeneration. Ragged red fibers were found in some areas. A second muscle biopsy at the age of 39 of the vastus lateralis muscle showed only minimal changes with mild predominance of type 2 fibers and mild variation of myofiber size. The patient continuously required NIV during the night but remained neurologically stable without progression of muscle weakness.


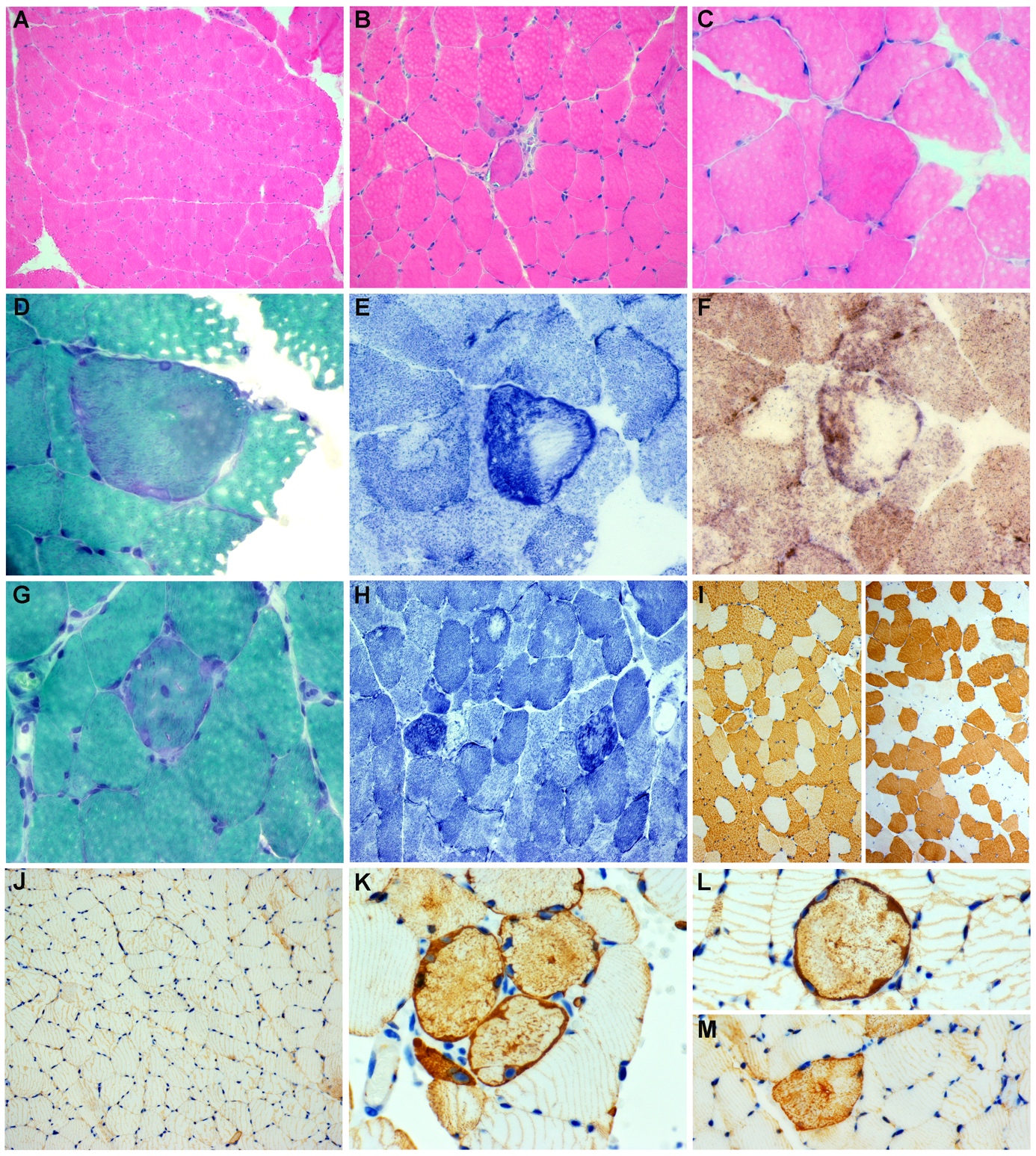


**Legend:** Myopathological findings of the muscle biopsy of the right quadriceps femoris muscle of patient #60 (*DNAJB4)*.

A: HE shows a slight variation of muscle fiber size with some small groups of moderately atrophic and angulated muscle fibers. B: Single muscle fiber necrosis and myophagias.

C: Some fibers show a subsarcolemmal accumulation of organelles. D: Gomori-Trichrome shows an unstructured muscle fiber with ill-defined amorphous material at the right. E: This region is devoid of NADH (E) and COX-SDH (F) activity. No COX negative fibers are detected. G: Another unstructured fiber with central nucleus with some features reminiscent of ragged-red fiber. H: NADH shows some muscle fibers with central irregularities and some moth-eaten pattern. I: Immunohistochemistry for fast (left) and slow (right) myosin shows that the small groups of moderately atrophic and angulated fibers correspond to type 2 fibers. J-M: Immunohistochemistry on paraffin tissue for desmin shows in J a globally preserved pattern. K-M depicts single fibers with enhanced subsarcolemmal accumulation of desmin and some irregular clumps, different from the pattern observed in regenerating fibers. *Original magnification:* *A, I x100. B, H, J: x 200. C, D, E, F, G, K: x400; L, M: x600*

## Cases with variants of uncertain significance (VUS)

**Patient #61** (*MARS*): This female patient first presented with a distal muscle weakness in lower limbs starting around the age of 24 years. Symptoms have resulted in marked gait difficulties that have progressed over time. On clinical examination, she had severe bilateral weakness of foot dorsiflexion (MRC 1-2) with distal atrophies. Deep tendon reflexes were absent. Sensory abnormalities included a reduced vibration sense. Nerve conduction studies (NCS) indicated a severe polyneuropathy (unable to differentiate between axonal and demyelinating). Nerve ultrasound showed no significant thickening. A thorough laboratory work-up including CSF analysis did not point toward an immunological cause.

**Patient #62** (*TRPV4*): This male individual initially developed weakness in his right arm when he was 44 years old. Symptoms have progressed rapidly, affecting all four limbs and requiring a tracheostomy due to respiratory insufficiency. On examination, he showed severe, distally pronounced tetraparesis, with the lower limbs being more severely affected (MRC 0-1), along with atrophies and fasciculations. Deep tendon reflexes were absent, and there were no sensory disturbances. NCS indicated motor neuropathy with axonal and demyelinating changes. Accordingly, neurogenic alterations were found on EMG. Muscle biopsy confirmed neurogenic changes. Genetic testing for 5q-SMA was negative. Over the past 15 years (since onset), the patient has remained stable using invasive ventilation and has experienced relapsing episodes of pneumonia.

**Patient #63** (*MYH2*): This gentleman has a progressive muscle weakness and muscle pain since age 40, causing impairments in daily life (standing up from a sitting position, climbing stairs, carrying weights, etc.). A neurological examination revealed mild ptosis, proximal atrophies of all four limbs with MRC 4. Deep tendon reflexes were reduced. CK levels were elevated 500-600 U/l. MRI of thigh muscles showed fatty atrophies, but no edema and no signs of inflammation. NCS/EMG indicated an underlying myopathic process. There is no cardiac involvement. The patient's father also experienced muscle weakness of unknown etiology since his fifties.

**Patient #64** (*BICD2*): This woman has experienced muscle weakness since birth. During childhood she was able to walk with crutches, but the progressive disease course with weakness and contractures in lower limbs has rendered her wheelchair dependent at the age of 16 years. In addition, she displays multiple orthopedic issues (scoliosis, hip dysplasia, contractures, etc.). On neurological examination in adulthood, she has mild weakness in upper limb muscles (MRC 4/4-), and more severe weakness in lower limbs (proximal MRC 2, distal MRC 4). Muscle tone and reflexes were reduced, and there were no sensory abnormalities. CK was mildly elevated (around 300-400 U/l). EMG showed neurogenic changes. Based on a previous muscle biopsy, central core disease was initially suspected. The family history was negative.

**Patient #65** (*TRPV4*): This man exhibited progressive lower motor neuron disease starting at the age of 68. Upon examination, he displayed symptoms such as dysarthria, dysphagia, fasciculations (observed in the tongue and thigh), and diffuse atrophies. Additionally, he experienced muscle weakness in the lower limbs (proximal MRC 4, right distal MRC 3-4, left distal MRC 4-5). There were no clinical indications of upper motor neuron involvement. Electromyography (EMG) revealed signs of denervation in 3 out of 4 levels. Brain and cervical spine MRI scans yielded normal results. As the disease progressed, he required non-invasive ventilation (NIV) and a percutaneous endoscopic gastrostomy (PEG) tube. He passed away 5 years after symptom onset.

**Patient #66** (*HNRNPA1*): This male patient has suffered from a neuromuscular condition since the age of 33. Initially, he displayed distal weakness in both upper limbs. Later on, he additionally developed gait problems. On neurological examination, he had severe, distally pronounced muscle atrophies. Weakness predominantly affected elbow extension (MRC 2-3) but also markedly impaired fine motor skills. In lower limbs, he also has mild proximal and severe distal weakness (MRC 1) on both sides. Sensory examination was largely normal. Reflexes were absent. Taken together, NCS, EMG and muscle biopsy all indicated a myopathy phenotype. The family history was negative.

**Patient #67** (*NIPA1*): This woman developed gait issues starting at the age of 52, partly accompanied by pain in lower limbs. Clinical examination revealed a spastic paraparesis with increased reflexes but no weakness. However, she had a mildly reduced vibration sense. MRI (brain and spine) did not reveal any explanatory results. DAT-SPECT was also normal; FDG-PET showed a reduced cortical uptake in the central region. The patient's mother had dementia (onset at 66 years).

**Patient #68** (*RYR1*): This young man has experienced multiple relapsing episodes of rhabdomyolysis following infections or physical activity (CK up to >11,000 U/l). Neurological examination is normal (between episodes). A muscle biopsy revealed no structural alterations, but a caffeine-halothane contracture test pointed towards malignant hyperthermia. The family history was negative.

**Patient #69** (*PYGM*): This male patient reported exercise intolerance since childhood with a positive "second wind" phenomenon. After long physical activity, he has muscle pain for up to a few days, sometimes accompanied by red urine. CK levels are elevated 1,500-3,000 U/l. Neurological examination was normal. Muscle MRI was normal.

**Patient #70** (*ACTN2*): This male patient first developed muscle cramps in his upper arms (asymmetric onset) at age 39. Moreover, he displays a mild proximal weakness in his lower extremities. He is only mildly affected and able to walk unaided. On neurological examination, an atrophy of the quadriceps femoris muscle was noted in addition to a mild weakness (MRC 4) for hip flexion. An EMG was normal. Muscle MRI of the lower limbs showed a mild, diffuse atrophy on the left when compared to the right side and a very mild edema affecting the extensor muscles, but no fatty degeneration. The family history is negative.

**Patient #71** (*REEP1*): This female patient presented with complaints of gait/balance issues and oscillopsia that began at the age of 50. Additionally, she exhibited atrophies in her right hand, resulting in reduced fine motor skills and mild weakness. NCS revealed a primary motor polyneuropathy with both axonal and demyelinating features. Video-nystagmography (VOG) confirmed bilateral vestibulopathy. Transcranial magnetic stimulation indicated a central lesion. MRI revealed mild vermian/cerebellar atrophy and the presence of a meningioma. Repeat expansions in RFC1 were found to be normal.

**Patient #72** (*SPAST*): This gentleman has a gait disorder starting around the age of 33 years. On examination, he exhibited a spastic paraparesis (mild proximal weakness, MRC 4) with brisk tendon reflexes and pyramidal tract signs bilaterally. Apart from lumbar disc herniations, an MRI of the entire spine and brain was normal. The family history was positive (mother and 2 children also affected).

**Patient #73** (*DMD*): This female patient had experienced moderate to severe psychomotor delay in early childhood, resulting in intellectual disability. Neurological examination revealed a mild proximal weakness in upper limbs (MRC 4), whereas lower limbs were more severely affected (MRC 3). She is able to walk a few steps. Muscle biopsy (performed in childhood) indicated a myopathic condition. Brain MRI showed no significant abnormalities. Muscle MRI (in adulthood) confirmed a severe fatty atrophy of nearly all investigated lower limb muscles (with the exception of the left semimembranosus muscle immediately proximal to the knee joint). The family history was negative.

**Patient #74** (*GAA*): This male individual showed symptoms onset at age 36. Before symptom onset, CK levels had already been elevated (800 U/l; later up to 2,000). Anti-Mi-2a antibodies were positive. On clinical examination, atrophy in both quadriceps femoris muscles and (pseudo)hypertrophy in both gastrocnemius muscles was found; the muscle tone was rather flaccid and a strength exam revealed MRC 4 for hip flexion, MRC 1 for knee extension on the right and 2-3 on the left side. Ankle dorsiflexion was preserved. Muscle MRI showed fatty atrophies with STIR hyperintensities and mild contrast enhancement. Muscle biopsy revealed myopathic alterations but also mild inflammatory infiltrations. Acid alpha glucosidase activity in leukocytes was reduced (1.1 μmol/L/h; reference >2).

**Patient #75** (*MPZ*): This female patient developed an episode of acute neuropathy (initially classified as Guillain-Barré syndrome) in her 50s. Due to symptoms progression (gait disorder) over the next 10 years, she was eventually diagnosed with CIDP. Clinically, she displayed distally pronounced sensory disturbances in both hands and feet. On examination, reduced vibration sense and gait imbalance were noted, but there was no circumscribed weakness. NCS showed marked (demyelinating > axonal) sensorimotor polyneuropathy. CSF analysis at disease onset was normal. Nerve ultrasound showed thickening of multiple peripheral nerves, which is in line with CIDP. A significant clinical response to IVIg has been documented in the past.

**Patient #76** (*MATR3*): This gentleman reported weakness for foot dorsiflexion (initially on the left, later on both sides) and occasional fasciculations since the age of 55. An initial neurological examination showed a bilateral foot drop (MRC 4) and comparably brisk tendon reflexes in lower limbs. During the further disease course, the patient also developed upper limb weakness with reduced fine motor skills and dysarthria/dysphonia and later also dysphagia. MRI of brain and cervical spine did not reveal explanatory results. An initial EMG showed signs of denervation in 1 of 4 levels, NCS did not indicate a polyneuropathy as underlying cause. IVIg trials did not show lasting responses. Clinically, the diagnosis of motor neuron disease was made.

**Patient #77** (*FIG4*): Following a thymectomy, this female patient developed progressive dysarthria, dysphonia, and hypersalivation with dysphagia at the age of 73. Although no localized weakness was observed, she experienced difficulties walking longer distances. NCS showed a mild axonal polyneuropathy, but EMG was entirely normal (no neurogenic changes, no signs of denervation). Also, brain and spinal MRI did not reveal any explanatory results. CSF analysis was unremarkable, serum NfL was increased (116 pg/ml; normal <45 pg/ml). Due to progressive bulbar symptoms, the patient eventually required a PEG. Hence, based on exclusion of alternative diagnoses, a motor neuron disorder was considered the most likely etiology.

**Patient #78** (*ITPR3*): This gentleman developed neuropathic pain/dysesthesias in both feet, especially when walking, since the age of 49 years. Symptoms have deteriorated over time. On examination, strength in distal leg muscles was mildly reduced (MRC 4). The ankle jerk reflex was absent on both sides, and vibration sense was also symmetrically reduced. NCS revealed a severe sensorimotor polyneuropathy. CSF analysis was normal. As comorbidities, the patient also suffers from depression and periodic limb movements during sleep.

**Patient #79** (*FA2H*): This man started to develop a slowly progressive gait disorder around the age of 50 years. Clinical examination revealed mild proximal weakness in lower limbs (MRC 4) and brisk deep tendon reflexes with increased tone. Brain MRI showed unspecific white matter lesions and atrophy of the superior cerebellar vermis. MRI of the spine did not indicate a myelopathy. The mother was also affected by a gait disorder, but did not undergo a systematic neurological assessment.

**Patient #80** (*FLNC*): This female patient has first experienced neuromuscular issues around 23 years of age. On neurological examination, she has bilateral foot drop (MRC 2) and absent deep tendon reflexes. CK was elevated (1000 U/l). EMG was clearly myopathic. Muscle MRI showed significant fatty atrophies in thigh and calf muscles with anterior and posterior compartments being involved. The family history was negative.

**Patient #81** (*FA2H*): This male patient had reported gait difficulties since the age 45 due to stiffness in the left leg. In addition, he has experienced difficulties writing as well as speech and memory problems. On examination, he has mild dysarthria, spasticity in lower limbs with increased tendon reflexes and pyramidal signs. An MRI of the brain revealed generalized atrophy, the spine was largely unremarkable. Transcranial magnetic stimulation indicated a central motor lesion. NCS were normal. The family history was negative.

**Patient #82** (*FA2H*): This male patient has developed a spastic paraparesis around the age of 29 years. MRI of the brain indicated mild cerebellar atrophy.

**Patient #83** (*TTN*): This gentleman developed a slowly progressive distal muscle weakness, initially affecting the left, and later also the right leg. On neurological examination, the patient had bilateral foot drop (MRC 0) with decreased tendon reflexes. EMG was myopathic. Muscle MRI of lower extremities showed symmetric fatty atrophies predominantly affecting extensor muscles. The father had been diagnosed with poliomyelitis.

## Cases in whom an acquired (non-monogenic) diagnosis was found after negative NGS.

**Patient #246:** This female patient first presented with distal sensory loss and muscle weakness at the age of 27. Nerve conduction studies showed a mixed (axonal-demyelinating) sensorimotor neuropathy (“strongly supportive of demyelination” according to the EAN/PNS 2021 criteria for CIDP). Repeated CSF examinations were normal, MRI showed thickening and contrast enhancement of lumbar nerve roots. The patient was treated with corticosteroids and IVIG and improved gradually but experienced multiple relapses during the course of disease which were successfully treated with plasma exchange and corticosteroids. *The final clinical diagnosis was distal chronic inflammatory demyelinating polyneuropathy.*

**Patient #247:** This 70-year-old man reported gait disturbances since approximately 33 years following a febrile infection. Neurological examination revealed spastic paraparesis, plantar responses were extensor. MRI showed atrophy and cystic defects in the cervical spinal cord and atrophy and T2 hyperintense lesion in the thoracic spinal cord. CSF was normal with negative oligoclonal bands. *The final clinical diagnosis was late sequela of post-infectious transverse myelitis.*

**Patient #248:** This male patient experienced progressive gait problems at the age of 62. Neurological examination revealed a spastic tetraparesis, deep tendon reflexes were increased at the knee and the ankle, plantar responses were extensor. MRI showed white matter lesions in the brain and cervical as well as thoracic spinal cord. Oligoclonal bands were positive in the CSF. *The final clinical diagnosis was primary progressive multiple sclerosis*.

**Patient #249:** This male patient first reported gait disturbances at the age of 52 with marked progression 3 years later. Clinically, he presented with a spastic ataxic gait, muscle tone was increased throughout (mildly in upper extremities and pronounced in lower extremities) , deep tendon reflexes were increased at the knee and ankle and plantar responses were positive. MRI showed cervical beginning cervical spondylotic myelopathy and the patient underwent decompressive surgery (anterior body fusion). *The final clinical diagnosis was cervical spondylotic myelopathy.*

**Patient #250:** This male patient reported neuropathic pain and distal sensory loss as well as mild distal weakness which started at the age of 46 in the left arm. Nerve conduction studies showed reduced sensory nerve action potential over the left median, ulnar and radial nerve. CSF was normal. MRI and ultrasound showed thickening and increased T2 signal intensity of the left brachial plexus without contrast enhancement. Biopsy of the left brachial plexus showed only fibrotic changes. The patient received treatment with IVIG, plasma exchange and corticosteroids without relevant improvement. *The final clinical diagnosis was sequela of left brachial plexus neuritis.*

**Patient #251:** This male patient presented with dysphagia, dysarthria and slowly progressive weakness and atrophy in the proximal more than the distal muscles starting approximately one year before at the age of 72. Deep tendon reflexes were absent throughout, sensory examination was normal. Nerve conduction studies showed axonal sensorimotor neuropathy, electromyography pathological spontaneous activity in 3 of 4 levels. Neurofilament light chain levels were within the normal range in serum and CSF, antibodies against gangliosides were negative. Muscle biopsy of the biceps femoris showed marked myopathic changes without inflammation, sural nerve biopsy axonal changes. Hematological evaluation including bone marrow biopsy led to the diagnosis of smoldering multiple myeloma. Because of neurological disease progression a therapy with daratumumab was started, which led to clinical stabilization of muscle weakness and improvement of bulbar signs. *The final clinical diagnosis was myopathy and neuropathy due to smoldering multiple myeloma.*

**Patient #252:** This female patient presented with progressive ataxic gait disturbances, cognitive deficits, muscle atrophy and weakness, dysarthria and distal sensory loss which started at the age of 59. Nerve conduction studies revealed sensorimotor axonal neuropathy, electromyography myogenic changes with pathological spontaneous activity and muscle biopsy of the lateral vastus myopathic changes compatible with metabolic or mitochondrial myopathies. Brain MRI showed mild iron accumulations in basal ganglia and CSF was normal. Because the patient had a history of multiple abdominal surgeries and a pathological hydrogen breath test, a diagnosis of bacterial overgrowth syndrome respectively malabsorption syndrome was suspected and laboratory tests showed severe reduction of Vitamin A, D, E and K. After high-dose vitamin supplementation the symptoms stabilized. *The final clinical diagnosis was severe deficiency of fat-soluble vitamins with neurological complications.*

**Patient #253:** This 42-year-old male patient noted slowly progressive speech and swallowing problems which plateaued after a few months. The patient had a history of nasopharyngeal carcinoma (epipharyngeal) which was treated with chemotherapy and local radiation 8 years earlier. Neurological examination revealed dysarthria and dysphagia with tongue atrophy and fibrillations. EMG and ultrasound showed fibrillations of the tongue but not in other muscles. MRI of the brain and neck showed post-actinic lipomatosis of cervical vertebrae 1-3 and the clivus but was unremarkable. *The final clinical diagnosis was delayed post-irradiation bulbar palsy (see e.g.: PMID: 11502928).*

**Patient #254:** This male patient presented because of slowly progressive spastic paraparesis with consecutive spastic-ataxic gait which he first noted at the age of 55. Clinically a gaze-evoked nystagmus, a skew deviation and mild reduced distal vibration sense was additionally found. MRI showed multiple infra-and supratentorial white matter lesions in the brain as well as one lesion in the cervical spinal cord. Oligoclonal bands were positive in the CSF. *The final clinical diagnosis was primary progressive multiple sclerosis.*

**Patient #255:** This 33-years old female patient had a history of t-cell lymphoma 6 years earlier. 2 years before presentation the patient noted progressive respiratory problems and was diagnosed with heart failure and cardiomyopathy which improved afterwards. Neurologically, she reported swallowing difficulties and a proximal muscle weakness in the shoulder girdle which started approximately 1 year ago. Electromyography revealed mild myogenic changes, MRI showed diffuse contrast enhancement, restricted diffusion and increased signal intensity of all shoulder muscles. Muscle biopsy showed muscle infiltration by mature t-cells (mostly CD8+ with partial CD7 loss). *The final clinical diagnosis was muscle infiltration by T cell large granular lymphocyte leukemia.*

**Patient #256:** This 21-year-old female patient reported progressive distal sensory loss and muscle weakness of the legs. Neurological examination revealed a fluctuating paraparesis, the muscle tone and deep tendon reflexes at the knee and ankle were initially graded as increased but were difficult to assess because of reduced patient cooperation, plantar responses were flexor. MRI of the brain and spinal cord was normal as was CSF examination. At a follow-up visit 6 months later, the symptoms of the patient had improved. *The final diagnosis was functional neurological disorder.*

**Patient #257:** This male patient reported that he was initially evaluated because of unilateral ptosis at the age of 16. In the following years he noted intermittent swallowing difficulties and slurred speech as well as fatigable muscle weakness. Additionally, he reported frequent diarrhea and excessive sweating. Anti-acetylcholine-receptor and anti-MuSK antibodies were negative as was repetitive nerve stimulation. The patient was treated with IVIG, corticosteroids and azathioprine, which mildly improved symptoms and underwent thymectomy. Reevaluation of the patient revealed mildly increased antibodies against N-type voltage gated calcium channels and the patient improved after 3,4-diaminopyridine was started. *The final clinical diagnosis was Lambert-Eaton myasthenic syndrome.*

**Patient #258:** This female patient reported distal sensory loss and pain in her fingers and toes that started at the age of 57. Nerve conduction studies revealed a demyelinating sensorimotor neuropathy (“strongly supportive of demyelination” according to the EAN/PNS CIDP 2021 criteria). Treatment with IVIG led to clinical improvement. *The final diagnosis was sensory predominant chronic inflammatory demyelinating neuropathy.*

**Patient #256:** This male patient developed proximal muscle weakness at the age of 65 following rhabdomyolysis due to high-dose atorvastatin. The patient improved following corticosteroid treatment. Muscle biopsy after corticosteroids were stopped was initially reported as compatible with metabolic myopathy. The patient remained initially stable with mild proximal muscle weakness and moderate CK elevation but experienced a relapse 4 years after the initial event. Clinical reevaluation including antibody testing revealed the presence of anti-HMGCR antibodies and the patient was successfully treated with IVIG. *The final clinical diagnosis was immune-mediated necrotizing myopathy with anti-HMGCR antibodies.*

**Patient #260:** This male patient first noted symptoms at the age of 16 with muscle weakness of the right finger extensors which gradually extended to more proximal muscles. At the age of 19 he developed similar muscle weakness in the left arm. Additionally, he noted fluctuating ptosis and double vision as well as fatigable muscle weakness. Repetitive nerve stimulation showed a decrement in all tested muscles, nerve conduction studies revealed pure motor demyelinating polyneuropathy. Anti-acetylcholine-receptor antibodies were positive, Anti-GM1-IgM antibodies negative. Repeated treatments with IVIG led to an improvement of myasthenic symptoms and mild improvement of muscle weakness. *The final clinical diagnosis was Anti-acetylcholine-receptor antibody early onset myasthenia gravis and immune-mediate pure motor asymmetric neuropathy (most likely multifocal motor neuropathy).*

# Supplementary tables

## Screened candidate genes

| **Gene** | **Disease phenotype** | **Inheri- tance** | **Allelic to OMIM#** |
| --- | --- | --- | --- |
| *﻿LAMA5* | Presynaptic congenital myasthenic syndrome | AR |  |
| *﻿UNC13A* | Presynaptic congenital myasthenic syndrome related to MUNC13-1 | AR |  |
| *﻿RPH3A* | Congenital myasthenic syndrome related to RPH3A | AR |  |
| *﻿TOR1AIP1* | Congenital myasthenic syndrome | AR | 617072 |
| *﻿CHD8* | Congenital myasthenic syndrome | AD | 615032 |
| *﻿ATP1A2* | Hypokalaemic periodic paralysis | AD | multiple (*182340) |
| *﻿TMEM65* | Mitochondrial myopathy with severe neurological manifestations | AR |  |
| *﻿TIMM22* | ?Combined oxidative phosphorylation deficiency 43 (#618851) | AR |  |
| *﻿APOO* | Mitochondrial myopathy with lactic acidosis, cognitive impairment and autistic features | XL |  |
| *﻿LRP10* | Amyotrophic lateral sclerosis | AD |  |
| *﻿SPTLC1* | AMYOTROPHIC LATERAL SCLEROSIS 27, JUVENILE; ALS27 (#620285) | AD |  |
| *﻿HEXB* | Late onset spinal muscular atrophy related to HEXB | AR | 268800 |
| *﻿PRUNE1* | Spinal muscular atrophy, related to PRUNE1 | AR | 617481 |
| *﻿KIF26B* | Spinal muscular atrophy with pontocerebellar hypoplasia related to KIF26B | AD |  |
| *﻿MAPT* | Lower motor neuron disease with respiratory failure related to MAPT | AD | multiple (*157140) |
| *﻿GOSR2* | Muscular dystrophy, congenital, with or without seizures (#620166) | AR | 614018 |
| *﻿CACNA1H* | Congenital amyotrophy | AR | multiple (*607904) |
| *﻿TNNC2* | Congenital myopathy 15 (620161) | AD |  |
| *﻿TRIM54+﻿TRIM63* | Cardiac and skeletal aggregate myopathy | digenic |  |
| *﻿PLIN4* | Distal Myopathy | AD |  |
| *﻿SYT2* | Distal motor neuropathy related to SYT2 | AD | multiple (*600104) |
| *﻿SPTAN1* | Distal motor neuropathy | AD | 613477 |
| *﻿PSAT1* | Progressive neuropathy | AR | 616038 and 610992 |
| *﻿ITPR3* | Charcot-Marie-Tooth disease, demyelinating, type 1J (#620111) | AD | 222100 |
| *CFAP276* | Charcot-Marie Tooth disease, intermediate | AD |  |
| *﻿AHNAK2* | Charcot-Marie Tooth disease | AR |  |
| *﻿KIF5A* | CMT2 related to KIF5A | AD | multiple (see *602821) |
| *﻿BAG3* | Charcot-Marie-Tooth disease, axonal, related to BAG3 | AD | 613881 and 612954 |
| *﻿SGPL1* | Charcot-Marie-Tooth disease, axonal | AR | 617575 |
| *﻿SCO2* | Charcot-Marie-Tooth disease, axonal, related to SCO2 | AR | 604377 and 608908 |
| *﻿SACS* | Charcot-Marie-Tooth disease, axonal; related to SACS | AR | 270550 |
| *﻿B4GALNT1* | Charcot-Marie-Tooth disease, axonal | AR | 609195 |
| *﻿NMNAT2* | Polyneuropathy with erythromelalgia | AR |  |
| *COQ7* | Distal hereditary motor neuropathy (PMID: 36454683) | AR | 616733 |
| *﻿SLC9A3R1* | Hereditary peripheral neuropathy | AD | 612287 |
| *TMEM63C* | Spastic paraplegia 87, autosomal recessive (619966) | AR |  |

**Supplementary table 1: Screened candidate genes.** List of candidate genes with disorders or phenotypes, which were not listed in OMIM at the time of reanalysis and were additionally screened. The disease name is based on the neuromuscular gene table if applicable (<https://www.musclegenetable.fr/>) and OMIM name and phenotype number is provided in cases that were already included in OMIM at time of publication. AD denotes autosomal dominant, AR autosomal recessive and XL X-linked.

## External genetic testing

| **Gene** | **Disorder** | **Negative (n)** | **Positive (n)** |
| --- | --- | --- | --- |
| *C9orf72* | Frontotemporal dementia and/or amyotrophic lateral sclerosis 1 | 56 | 0 |
| *SMN1* | Spinal muscular atrophy (1-4) | 7 | 2 |
| *DMPK* | Myotonic dystrophy 1 | 6 | 0 |
| *CNBP* | Myotonic dystrophy 2 | 5 | 1 |
| *AR* | Spinal and bulbar muscular atrophy of Kennedy | 6 | 0 |
| *DUX4 (D4Z4)* | Facioscapulohumeral muscular dystrophy 1 | 4 | 0 |
| *Ataxia panels* | Spinocerebellar ataxias and Friedrich ataxia | 8 | 0 |

**Supplementary table 2: External genetic testing.** 81 patients were externally tested for disorders not covered by our short-read NGS approach which solved 3 additional cases.

## Sequencing details

|  | **All patients (n=263)** | **Patients with a molecular diagnosis (n=60)** | **Patients without a molecular diagnosis (n=203)** | **p-value** |
| --- | --- | --- | --- | --- |
| Median coverage WES (IQR) | 105 (89-127) | 110 (93.5-132) | 104 (88-126.5) | 0.24 |
| Median coverage WGS (IQR) | 52 (50-53) | 50.5 (50-51) | 52 (50-53) | 0.57 |
| Sequencer (Illumina) |  |  |  | 0.05^§^ |
| HiSeq2500 | 17 (6%) | 6 (10%) | 11 (5%) |  |
| HiSeq4000 | 84 (32%) | 25 (42%) | 59 (29%) |  |
| HiSeq6000 | 162 (62%) | 29 (48%) | 133 (66%) |  |
| Enrichment Kits WES |  |  |  | 0.34 |
| SureSelect Human All     Exon Kit (Agilent,     50mb V5) | 17 (7%) | 6 (10%) | 11 (5%) |  |
| SureSelect Human All     Exon Kit (Agilent 60mb     V6) | 178 (73%) | 43 (72%) | 135 (67%) |  |
| Twist Human Exome     2.0 Plus     Compreh-ensive     Exome Spike-in and     Mitochondrial Panel | 50 (20%) | 9 (15%) | 41 (20%) |  |
| Enrichment Kit WGS |  |  |  | NA |
| Illumina DNA     PCR-Free Library     Preparation Kit) | 18 (100%) | 2 (3%) | 16 (8%) |  |

**Supplementary table 3: External genetic testing.** Sequencing details for all patients as well as for patients with a molecular diagnosis compared to patients without a molecular diagnosis after NGS. IQR denotes interquartile range, WES whole-exome sequencing and WGS whole-genome sequencing.

§ statistically significant
